# Supplementary material for: Influence of Light of Different Spectral Compositions on Growth Parameters, Photosynthetic Pigment Contents and Gene Expression in Scots Pine Plantlets
Source: Int J Mol Sci. 2023 Jan 20;24(3):2063. doi: 10.3390/ijms24032063 (PMC9917036; doi:10.3390/ijms24032063)
Supplement: Supplementary file 1 [file ijms-24-02063-s001.zip › ijms-2111969-supplementary.pdf]

Table S1. Transcript levels of main photoreceptors and transcription factors involved in light signaling in Scots pine plantlets under different light quality and dark conditions.

Light	Days	Photoreceptors					TFs	
		PHYP	*PHYO*	*PHYN*	*CRY1*	*CRY2*	*HY5*	*PIF3*
WFL	0	1.23±0.07 a	1.02±0.05 a	2.37±0.08 a	2.05±0.06 a	0.45±0.04 b	1.36±0.07 b	4.37±0.27 *a*
	1	0.46± 0.04 b	0.62±0.02 b	1.04±0.05 b	1.57±0.06 c	0.46±0.03 b	1.08±0.08 b	2.25±0.09 *ab*
	3	0.41±0.05 bc	0.51±0.02 b	0.62±0.05 c	0.88±0.05 e	0.34±0.03 b	1.08±0.08 b	2.49±0.24 *ab*
	5	0.28±0.02 c	1.12±0.07 a	0.76±0.04 c	1.08±0.04 d	1.13±0.07 a	1.18±0.10 b	2.24±0.21 *ab*
	7	0.26±0.02 c	0.22±0.02 c	0.12±0.01 d	1.76±0.04 b	0.35±0.03 b	2.06±0.12 a	0.74±0.20 *c*
	9	0.35±0.02 bc	0.23±0.03 c	0.11±0.01 d	2.18±0.03 a	0.32±0.02 b	2.15±0.09 a	1.33±0.12 *b*
WL	0	1.21±0.03 a	0.61±0.05 c	2.31±0.11 a	1.74±0.04 a	1.15±0.05 *a*	1.04±0.05 c	1.31±0.04 a
	1	0.65±0.02 b	0.67±0.04 c	1.16±0.05 c	0.50±0.05 c	0.67±0.05 *a*	1.68±0.06 b	1.26±0.04 a
	3	0.58±0.02 b	1.02±0.05 a	1.38±0.05 b	0.93±0.03 b	1.05±0.05 *a*	1.72±0.03 b	1.27±0.04 a
	5	0.52±0.03 c	0.85±0.04 b	0.33±0.04 d	0.94±0.02 b	1.11±0.05 *a*	1.99±0.08 a	1.19±0.03 a
	7	0.44±0.01 d	0.87±0.03 b	0.16±0.04 d	0.54±0.05 c	1.16±0.06 *a*	1.58±0.06 b	1.21±0.04 a
	9	0.36±0.02 e	0.77±0.05 b	0.27±0.05 d	1.00±0.08 b	0.67±0.05 *a*	1.07±0.04 c	1.04±0.05 b
BL	0	0.32±0.01 c	0.68±0.05 c	2.51±0.03 a	2.25±0.04 b	2.03±0.04 *a*	1.75±0.04 a	1.20±0.16 c
	1	0.57±0.05 b	0.62±0.04 c	2.19±0.04 b	0.88±0.06 d	0.85±0.05 *ab*	1.44±0.04 b	1.16±0.13 cd
	3	0.58±0.01 b	1.44±0.05 a	0.65±0.04 d	0.82±0.04 d	0.87±0.05 *ab*	1.81±0.03 a	0.62±0.16 d
	5	1.07±0.03 a	1.15±0.05 b	1.11±0.07 c	0.77±0.03 d	0.60±0.04 *b*	1.34±0.06 bc	1.48±0.25 bc
	7	0.53±0.08 b	0.76±0.04 c	0.76±0.03 d	1.14±0.04 c	0.85±0.05 *ab*	1.06±0.07 d	1.92±0.04 b
	9	0.39±0.03 c	1.07±0.05 b	0.73±0.04 d	2.53±0.03 a	1.05±0.04 *a*	1.25±0.05 c	2.73±0.22 a
RL	0	0.30±0.03 d	0.81±0.03 c	2.79±0.06 a	1.04±0.04 c	1.24±0.06 b	0.98±0.08 a	0.87±0.04 c
	1	0.88±0.11 c	0.75±0.06 c	1.86±0.04 b	1.57±0.07 b	2.02±0.06 a	1.22±0.05 a	1.13±0.08 c
	3	1.28±0.06 b	1.23±0.04 a	1.84±0.06 b	1.45±0.05 b	2.14±0.07 a	1.08±0.07 a	1.49±0.21 b
	5	1.19±0.04 b	0.77±0.04 c	1.68±0.13 b	2.08±0.03 a	2.19±0.18 a	1.16±0.07 a	2.11±0.03 a
	7	1.26±0.03 b	1.14±0.06 ab	1.03±0.06 c	1.13±0.07 c	1.05±0.05 b	1.23±0.08 a	1.24±0.05 bc
	9	1.56±0.03 a	1.05±0.04 b	0.78±0.05 d	1.54±0.05 b	1.19±0.02 b	1.05±0.10 a	1.29±0.05 bc
FRL	0	1.28±0.06 a	1.45±0.04 *c*	2.57±0.20 a	0.57±0.04 c	1.03±0.05 b	0.98±0.09 c	0.30±0.05 e
	1	0.51±0.06 c	1.78±0.06 *ab*	2.33±0.05 a	0.75±0.05 b	1.32±0.05 a	1.50±0.01 b	0.54±0.03 d
	3	0.77±0.02 bc	1.78±0.04 *ab*	0.65±0.07 d	0.69±0.02 bc	0.83±0.06 c	0.57±0.06 d	0.61±0.02 d
	5	1.15±0.05 a	1.58±0.02 *b*	1.75±0.05 b	1.06±0.05 a	1.08±0.06 b	1.06±0.05 c	1.08±0.05 c
	7	0.66±0.04 c	2.49±0.19 *a*	0.95±0.08 c	0.74±0.06 b	1.00±0.07 b	1.74±0.04 a	1.78±0.06 a
	9	0.83±0.07 b	1.65±0.04 *b*	1.05±0.05 c	1.12±0.02 a	0.72±0.02 c	0.97±0.07 c	1.43±0.06 b
Dark	0	0.12±0.03 b	0.11±0.00 *b*	2.28±0.13 a	1.03±0.07 c	0.53±0.05 bc	0.32±0.02 d	1.00±0.12 e
	1	0.09±0.03 b	0.11±0.00 *b*	2.46±0.13 a	1.22±0.01 b	1.14±0.09 a	0.17±0.04 e	2.29±0.11d
	3	0.14±0.02 b	0.12±0.00 *b*	2.51±0.11 a	2.08±0.08 a	0.65±0.04 b	0.15±0.03 e	3.73±0.29 c
	5	0.14±0.03 b	0.73±0.06 *b*	1.15±0.15 b	0.63±0.06 d	0.46±0.03 c	1.06±0.07 b	4.80±0.40 b
	7	1.11±0.09 a	1.61±0.13 *a*	2.39±0.16 a	1.06±0.05 bc	0.58±0.03 bc	0.93±0.03 c	5.73±0.27 a
	9	0.16±0.03 b	0.42±0.05 *b*	2.64±0.13 a	0.58±0.05 d	0.50±0.01 bc	1.26±0.03 a	0.68±0.10 e

Different letters denote statistically significant differences in the means at $p < 0.05$ (ANOVA followed by Duncan's method) between the experimental treatments. Different italic letters denote statistically significant differences in the means at $p < 0.05$ (Kruskal–Wallis ANOVA of the ranks followed by the Student-Newman–Keuls post hoc test) between the experimental treatments.

Table S2. Transcript levels of main genes of light harvesting complex of Chl *a*/*b* binding proteins, and large subunit of RubisCo, chlorophyll biosynthesis gene, main gene of carotenoids biosynthesis in Scots pine plantlets under different light quality and dark conditions.

**Light**	**Days**	**Photosystems**			**Chlorophyll**	**Carotenoids**
		LHCa1	*LHCb2*	*rbcL*	*PORA*	*PSY*
WFL	0	0.44±0.06 d	0.54±0.07 e	0.96±0.04 d	1.38±0.07 cd	0.65±0.04 *b*
	1	1.17±0.09 c	1.72±0.10 c	0.85±0.07 d	2.21±0.19 b	1.18±0.06 *a*
	3	1.13±0.08 c	1.41±0.03 c	0.44±0.06 e	1.31±0.08 d	0.98±0.07 *a*
	5	0.93±0.04 c	1.23±0.05 d	2.56±0.21 c	1.74±0.07 c	1.15±0.04 *a*
	7	2.86±0.07 b	3.51±0.22 b	2.92±0.03 b	1.38±0.03 c	1.34±0.05 *a*
	9	3.67±0.18 a	4.27±0.08 a	3.30±0.14 a	2.78±0.22 a	1.56±0.28 *a*
WL	0	0.66±0.05 e	0.86±0.04 d	0.53±0.06 c	1.21±0.04 *d*	0.59±0.13 c
	1	0.55±0.04 e	1.26±0.05 d	0.65±0.06 bc	1.24±0.04 *d*	1.12±0.10 b
	3	1.35±0.04 d	2.76±0.13 c	0.61±0.04 c	1.14±0.07 *d*	1.08±0.12 b
	5	2.89±0.03 c	2.97±0.11 c	0.89±0.05 b	2.62±0.19 *c*	1.05±0.15 b
	7	3.13±0.01 b	3.48±0.24 b	0.80±0.09 bc	2.97±0.01 *b*	1.11±0.13 b
	9	3.79±0.10 a	4.42±0.25 a	2.67±0.14 a	3.13±0.04 *a*	2.41±0.21 a
BL	0	0.18±0.02 d	0.16±0.03 *c*	1.07±0.05 c	1.70±0.07 b	0.33±0.05 *d*
	1	0.36±0.04 c	0.54±0.07 *bc*	0.61±0.05 d	1.45±0.05 bc	0.48±0.02 *c*
	3	0.66±0.04 b	0.59±0.05 *bc*	1.16±0.05 c	2.55±0.16 a	1.23±0.15 *bc*
	5	0.79±0.02 b	0.85±0.03 *b*	1.04±0.05 c	1.25±0.04 c	1.10±0.07 *bc*
	7	0.73±0.06 b	0.78±0.03 *b*	1.48±0.06 b	1.52±0.10 bc	1.50±0.12 *b*
	9	3.02±0.06 a	3.17±0.14 *a*	3.12±0.08 a	1.08±0.05 c	2.62±0.38 *a*
RL	0	0.26±0.03 d	0.25±0.03 d	0.41±0.04 f	1.24±0.05 d	0.38±0.02 d
	1	0.86±0.06 c	1.02±0.06 c	1.50±0.09 d	3.12±0.09 b	0.74±0.08 c
	3	1.15±0.04 b	1.40±0.07 b	1.12±0.09 e	2.41±0.06 c	1.07±0.06 b
	5	1.17±0.07 b	1.01±0.09 c	2.64±0.06 a	4.20±0.16 a	2.24±0.03 a
	7	1.20±0.03 b	1.12±0.07 c	2.25±0.13 b	1.25±0.09 d	1.19±0.07 b
	9	2.96±0.10 a	2.86±0.07 a	1.92±0.08 c	0.45±0.03 e	1.25±0.07 b
FRL	0	0.22±0.03 e	0.20±0.05 e	0.29±0.02 c	0.88±0.06 b	0.18±0.02 d
	1	0.75±0.06 d	0.55±0.05 d	0.56±0.06 c	1.21±0.06 a	0.66±0.03 b
	3	0.97±0.08 c	1.00±0.07 c	1.02±0.06 b	0.85±0.05 b	0.49±0.04 c
	5	1.09±0.06 c	1.13±0.03 c	1.02±0.08 b	1.14±0.05 a	0.65±0.02 b
	7	3.12±0.08 a	2.78±0.17 a	1.28±0.04 b	1.16±0.04 a	0.80±0.02 a
	9	1.71±0.04 b	1.74±0.04 b	1.62±0.20 a	0.65±0.05 c	0.78±0.03 a
Dark	0	0.85±0.04 c	0.64±0.06 *a*	0.85±0.04 d	0.86±0.04 *c*	0.49±0.03 b
	1	1.27±0.03 a	0.44±0.07 *a*	1.52±0.05 a	1.05±0.05 *b*	0.44±0.01 bc
	3	1.06±0.06 b	0.56±0.06 *a*	1.25±0.05 b	1.13±0.07 *b*	0.45±0.02 bc
	5	0.90±0.05 c	1.03±0.07 *a*	0.46±0.05 f	1.26±0.05 *b*	0.41±0.01 c
	7	1.15±0.05 ab	0.99±0.08 *a*	0.66±0.04 e	1.64±0.05 *a*	0.55±0.02 a
	9	1.14±0.04 ab	1.03±0.05 *a*	1.08±0.05 c	1.74±0.07 *a*	0.28±0.01 d

Different letters denote statistically significant differences in the means at $p < 0.05$ (ANOVA followed by Duncan's method) between the experimental treatments. Different italic letters denote statistically significant differences in the means at $p < 0.05$ (Kruskal–Wallis ANOVA of the ranks followed by the Student-Newman–Keuls post hoc test) between the experimental treatments.

Table S3. Transcript levels of genes of gibberellic acid biosynthesis and signaling, as well as ABA biosynthesis and signaling, main genes of auxin signaling, cytokinin signaling and salicylic acid signaling gene, jasmonic acid signaling gene in Scots pine plantlets under different light quality and dark conditions.

**Light**	**Days**	**Gibberellic acid**				**Abscisic acid**			**Auxins**	**Cytokinins**		**Salicylic acid**	**Jasmonates**
		GA3ox1	*GA3ox2*	*KAO1*	*KAO2*	*NCED*	*PYR*	*CCH1*	*AUX/IAA*	*HPT1*	*RRa*	*PR1*	*JazA*
WFL	0	0.14±0.04c	0.15±0.03*b*	0.45±0.07d	0.23±0.03e	0.18±0.02d	1.59±0.16a	1.26±0.08b	0.61±0.03bc	1.87±0.12b	0.14±0.02*c*	0.25±0.01c	2.28±0.10b
	1	0.20±0.05c	1.49±0.23*a*	0.83±0.12cd	1.22±0.04d	1.20±0.09c	0.87±0.05b	0.22±0.02c	0.72±0.08b	0.93±0.07c	0.18±0.01*c*	0.27±0.02c	3.48±0.15a
	3	0.17±0.04c	0.46±0.08*b*	0.48±0.09d	1.65±0.06c	0.40±0.03d	0.34±0.05c	0.53±0.09c	0.43±0.08bc	0.85±0.14cd	0.14±0.02*c*	0.23±0.01c	0.55±0.08d
	5	0.19±0.05c	1.05±0.08*a*	1.06±0.08c	1.13±0.08d	1.17±0.09c	0.82±0.06b	1.24±0.14b	1.87±0.19a	2.38±0.20a	0.33±0.04*c*	0.23±0.01c	1.00±0.13c
	7	3.73±0.32b	0.24±0.05*b*	3.73±0.25a	2.78±0.17b	3.19±0.30b	0.79±0.11b	1.66±0.10b	0.34±0.01c	0.78±0.02cd	2.66±0.16*a*	3.08±0.08a	0.89±0.08d
	9	6.17±0.55a	0.18±0.02*b*	2.81±0.16b	3.57±0.24a	3.84±0.23a	0.39±0.08c	3.01±0.36a	0.35±0.02c	0.50±0.03d	1.97±0.12*b*	1.71±0.10b	2.47±0.18b
WL	0	0.14±0.04c	0.21±0.05c	0.55±0.07d	0.21±0.05d	0.12±0.01b	1.35±0.07c	0.22±0.05c	1.23±0.03*c*	0.55±0.06*e*	0.63±0.06e	0.24±0.02e	0.56±0.05c
	1	0.20±0.04c	0.09±0.01c	2.75±0.30b	0.75±0.03c	0.17±0.05b	2.16±0.12b	2.44±0.15a	1.02±0.05*d*	0.74±0.04*d*	1.52±0.06c	0.62±0.05d	0.86±0.04b
	3	0.13±0.03c	0.17±0.03c	1.98±0.26c	0.83±0.05c	0.21±0.01b	0.98±0.06d	2.14±0.09a	1.44±0.04*b*	1.63±0.08*b*	1.76±0.04b	1.73±0.05b	2.31±0.11a
	5	0.13±0.02c	3.76±0.44a	3.25±0.13b	2.91±0.18a	0.19±0.02b	3.20±0.17a	2.64±0.28a	1.39±0.05*b*	0.95±0.02*c*	2.33±0.06a	2.54±0.10a	0.42±0.04c
	7	2.12±0.09a	2.07±0.08b	4.58±0.34a	2.39±0.20b	0.23±0.02b	3.04±0.11a	2.33±0.11a	2.23±0.18*a*	1.83±0.03*a*	1.02±0.07d	2.45±0.06a	0.15±0.03d
	9	1.05±0.09b	0.18±0.03c	1.05±0.09d	0.94±0.03c	1.08±0.10a	0.23±0.08e	1.15±0.18b	0.91±0.03*e*	1.12±0.08*c*	1.20±0.06d	1.08±0.05c	0.18±0.02d
BL	0	0.11±0.01*bc*	0.74±0.03b	0.66±0.04d	0.44±0.06d	0.15±0.02c	1.73±0.05a	0.80±0.09c	0.33±0.08d	0.87±0.07cd	1.44±0.04b	0.24±0.03b	0.44±0.03bc
	1	0.07±0.03*c*	0.42±0.06c	0.64±0.05d	0.61±0.05d	0.17±0.01c	0.17±0.04b	0.84±0.06c	0.99±0.05c	1.26±0.04b	1.48±0.02b	0.35±0.04b	0.67±0.06b
	3	0.23±0.03*b*	0.19±0.02d	1.11±0.06c	1.32±0.05b	0.23±0.03c	0.16±0.03b	1.11±0.07c	0.42±0.06d	0.76±0.04d	1.79±0.03a	0.48±0.03b	0.51±0.05bc
	5	0.14±0.03*bc*	1.03±0.07a	1.04±0.03c	1.06±0.07c	1.03±0.07b	0.20±0.02b	1.02±0.06c	1.67±0.10b	1.03±0.06c	0.95±0.03c	0.46±0.04b	0.44±0.02bc
	7	0.17±0.04*bc*	0.20±0.01d	2.24±0.13b	1.75±0.06a	1.09±0.07b	0.17±0.03b	2.06±0.08b	0.55±0.04d	1.26±0.04b	1.42±0.05b	0.25±0.02b	0.41±0.11c
	9	2.74±0.31*a*	0.28±0.03d	2.66±0.28a	1.31±0.06b	2.39±0.21a	0.21±0.03b	2.44±0.24a	2.65±0.08a	2.12±0.11a	1.82±0.05a	3.15±0.10a	4.28±0.12a
RL	0	0.09±0.04b	0.17±0.03c	1.06±0.10b	0.17±0.03d	0.11±0.03a	1.92±0.19a	0.17±0.03b	1.36±0.04d	0.75±0.06e	1.10±0.14c	0.30±0.03d	4.23±0.28*b*
	1	0.16±0.04b	0.16±0.04c	0.35±0.05b	0.63±0.05c	0.13±0.04a	0.48±0.09b	0.16±0.04b	3.29±0.17a	2.39±0.10a	0.77±0.05c	3.79±0.17a	6.35±0.71*a*
	3	0.11±0.05b	2.49±0.27b	0.52±0.06b	0.48±0.08c	0.18±0.02a	0.16±0.06c	0.11±0.00b	2.42±0.08b	1.64±0.06b	1.50±0.12b	0.84±0.07c	4.89±0.20*b*
	5	0.10±0.02b	4.26±0.28a	3.83±0.30a	1.31±0.07b	0.12±0.05a	0.25±0.04bc	0.15±0.01b	1.16±0.06d	1.11±0.09d	0.66±0.10d	0.74±0.08c	3.14±0.11*c*
	7	0.18±0.04b	0.50±0.12c	4.61±0.52a	1.50±0.08a	0.30±0.05a	0.23±0.05bc	2.58±0.23a	1.11±0.06d	1.19±0.04c	2.28±0.17a	1.05±0.05bc	1.02±0.05*e*
	9	0.44±0.06a	0.19±0.05c	3.83±0.28a	1.62±0.07a	0.19±0.04a	0.19±0.04bc	2.98±0.37a	1.65±0.08c	1.35±0.05c	1.67±0.09b	1.34±0.09b	2.30±0.20*d*
FRL	0	0.04±0.02a	0.11±0.05a	0.63±0.06c	0.07±0.03a	0.16±0.03a	1.40±0.12a	0.44±0.05c	0.42±0.06c	0.66±0.04e	0.25±0.04c	0.19±0.04e	1.35±0.05a
	1	0.12±0.05a	0.33±0.14a	2.66±0.18b	0.16±0.07a	0.08±0.04a	0.75±0.04b	0.43±0.06c	1.13±0.06b	1.25±0.03c	2.01±0.10a	0.64±0.04c	0.73±0.22b
	3	0.13±0.06a	0.17±0.03a	2.15±0.08b	0.13±0.02a	0.21±0.10a	0.52±0.07bc	1.35±0.05b	1.01±0.07b	0.95±0.03d	0.46±0.03b	1.11±0.06b	1.05±0.06ab
	5	0.10±0.05a	0.15±0.03a	2.38±0.22b	0.15±0.03a	0.11±0.05a	0.38±0.03c	1.25±0.05b	1.27±0.03ab	1.26±0.04c	0.23±0.02c	0.56±0.04cd	0.63±0.06b
	7	0.06±0.04a	0.16±0.06a	4.14±0.31a	0.22±0.03a	0.12±0.05a	1.52±0.14a	1.11±0.08b	1.17±0.05b	2.48±0.16a	0.15±0.03c	1.77±0.08a	0.47±0.11b
	9	0.10±0.04a	0.14±0.04a	4.11±0.28a	0.20±0.04a	0.09±0.04a	0.62±0.03bc	1.60±0.13a	1.41±0.05a	1.50±0.09b	0.15±0.06c	0.41±0.06d	0.56±0.02b
Dark	0	0.06±0.03a	0.18±0.02a	0.07±0.01d	0.10±0.05a	0.16±0.03a	1.83±0.14b	1.31±0.11e	0.77±0.06d	1.85±0.04c	0.36±0.04c	0.24±0.02c	0.34±0.04*d*
	1	0.07±0.02a	0.17±0.03a	0.18±0.01d	0.21±0.04a	0.14±0.07a	0.27±0.03d	2.12±0.12d	2.58±0.20a	3.16±0.10a	0.85±0.04b	0.71±0.07b	3.85±0.43*a*
	3	0.11±0.06a	0.20±0.04a	2.16±0.18a	0.14±0.02a	0.04±0.03a	3.26±0.28a	5.44±0.38a	1.99±0.08b	2.55±0.03b	0.57±0.05c	0.21±0.00c	2.36±0.06*b*
	5	0.11±0.05a	0.14±0.04a	1.00±0.06c	0.16±0.07a	0.09±0.07a	1.04±0.09c	3.75±0.19c	1.04±0.05cd	1.00±0.06e	0.69±0.07bc	0.58±0.06b	1.00±0.10*cd*
	7	0.11±0.04a	0.34±0.06a	1.29±0.15bc	0.14±0.07a	0.13±0.06a	1.12±0.07c	4.62±0.32b	1.32±0.04c	1.34±0.03d	1.49±0.13a	0.47±0.08bc	1.11±0.06*cd*
	9	0.10±0.05a	0.13±0.06a	1.46±0.13b	0.09±0.01a	0.09±0.04a	2.14±0.11b	3.52±0.31c	1.75±0.13b	1.96±0.07c	0.65±0.07bc	2.17±0.18a	1.35±0.03*c*

Different letters denote statistically significant differences in the means at $p < 0.05$ (ANOVA followed by Duncan's method) between the experimental treatments. Different italic letters denote statistically significant differences in the means at $p < 0.05$ (Kruskal–Wallis ANOVA of the ranks followed by the Student-Newman–Keuls post hoc test) between the experimental treatments.

Table S4. List of primers used for qRT-PCR analysis

№	Gene Bank ID	Gene	Description	Primer 5′-3′	
1	ALN42232.1 (uniprot.org)	*HPT1*	Histidine-containing phosphotransfer 1	GCTCAAGTATAGGAGCGCGG	CCAGCTTGTTTTCACGAGGT
2	FJ717710.1 (ncbi.nlm.nih.gov)	*RRa*	Type-A Response Regulators	CAGAAGGCGCTCAAGAGTTT*	TTGTTGGTCCCTGGATCTTC*
3	EF083399.1 (ncbi.nlm.nih.gov)	*JazA*	Jasmonate-Zim domain 1	GGTGAACGTGTATGATGATATTCC	CGTTGCAGAGAATGCTTCCTC
4	EF084624.1 (ncbi.nlm.nih.gov)	*PR1*	Pathogenesis-related protein 1	GCACTCTGGTGGTCAATACG	CACCCGAGCCTCTTGGAA
5	D5ABG4 (uniprot.org)	*PIF3*	Phytochrome-interacting factor 3	ATCAGCACTTCCTGGTTCCG	CAGGCTGAGTTGTTCCAGGT
6	Z16409.1 (uniprot.org)	*LHCa1*	Chlorophyll a-b binding protein, chloroplastic	CCAGCATTGGCCTGATCAAC	CCACTTCGTTGGGAGGAAGA
7	AAB19040.1 (uniprot.org)	*LHCb2*	Chlorophyll a-b binding protein, chloroplastic	TGCCAAGAACAGAGAGCTGG	ATGCTCTGGGCGTGAATCAA
8	O22599 (uniprot.org)	*PORA*	NADPH-protochlorophyllide oxidoreductase PORA	ACTACTGGACTCTTCCGCGA	GTGCAAGCCTTTTTCCTGCT
9	AY289600.1 (ncbi.nlm.nih.gov)	*AUX/IAA*	Auxin-induced protein 1 (IAA1)	GCCACCTGTCAAAGATTTCAG	TGAGGTCCACCTTTCTGAGA
10	MA_10174788g0010 (congenie.org)	*NCED*	9-cis-epoxycarotenoid dioxygenase	GCAAGGCATCGCCAGCTA	CCCGAAGAGGCTTCTTGCA
11	MA_10302927g0020 (congenie.org)	*PYR*	Abscisic acid receptor PYR	GAAGGTTGTTTGGCTCTCCG	TCTCCGAACCACTGACCACA
12	A9NTI7 (uniprot.org)	*HY5*	BZIP domain-containing protein Transcriptional factor HY5	ACACAGCATCAGTCCACAAGT	GTAGATGGCCCTGCTTGCAT
13	MA_108278g0010 (congenie.org)	*CCH1*	ABA-binding protein Chloroplast Mg^{2+}-chelatase/ABA Receptor	AAATCAGGGTCTGCCCAGTG	TGGATTGGCCCAACTGAGAC
14	K7R334 (uniprot.org)	*CRY1*	Cryptochrome 1	TATGGTGCACAGGGCAGATG	AAGCTGCAGAAGCTGTTCCT
15	T2FFB6 (uniprot.org)	*CRY2*	Cryptochrome 2	TTCCCTGGCTGCAACAGAAA	CCCAACATTGCTAGGCAGGA
16	AIY54822.1 (uniprot.org)	*PHYP*	Phytochrome P	GGCATGTCCCTTGTTCAGGA	CTTCTGTGGGCCAAAGGTCT
17	AFV79519.1 (uniprot.org)	*PHYN*	Phytochrome N	GGCTCAGAGGAGGACAAAGG	TTCTGCCCGGTCACATCTTG
18	A7Y6Q6 (uniprot.org)	*PHYO*	Phytochrome O	AGATGTGACGTGGCAAAGGA	TGCGGGATTCCACTCAGAAC
19	AET45533.1 (uniprot.org)	*rbcL*	RuBisCO large chain	CAACCATTCATGCGCTGGAG	TGCGGTAAAACCTCCCGTC
20	MA_407452g0010 (congenie.org)	*PSY*	Phytoene synthase	TCAAGATGAGCTTGCACGGA	ACCGGCCATCTACTGGTTTT
21	X5CFJ2 (uniprot.org)	*GA3ox1*	Gibberellin 3-beta-dioxygenase 1	CAGAAGCAGAAGCAGACTTT	ATAACAGTGGATGCTTGGAAT
22	X5CWJ3 (uniprot.org)	*GA3ox2*	Gibberellin 3-beta-dioxygenase 2	GCATCAAGCCCAGACTTT	TATAAGAGAGGGTGCTTGGAAT
23	X5CHS9 (uniprot.org)	*KAO1*	Ent-kaurenoic acid oxidase 1	TACAGGTGGTGGAATGCTC	CTGTAGGTTTCGCCAATCAG
24	X5CAD0 (uniprot.org)	*KAO2*	Ent-kaurenoic acid oxidase 2	AAATGCTGGGCATGAGTC	CGATCTATCCTTCTCTTCACAA
25	CBB44933.1 (uniprot.org)	*ACT1*	Actin 1	TTAGCAACTGGGATGACATGGA	CCTGAATGGCAACATACATAGCA
